# Realizing depth measurement and edge detection based on a single metasurface

**DOI:** 10.1515/nanoph-2023-0308

**Published:** 2023-07-12

**Authors:** Siwen Yang, Qunshuo Wei, Ruizhe Zhao, Xin Li, Xue Zhang, Yao Li, Junjie Li, Xiaoli Jing, Xiaowei Li, Yongtian Wang, Lingling Huang

**Affiliations:** Beijing Engineering Research Center of Mixed Reality and Advanced Display, School of Optics and Photonics, Beijing Institute of Technology, Beijing, 100081, China; Beijing National Laboratory for Condensed Matter Physics, Institute of Physics, Chinese Academy of Sciences, Beijing, 100190, China; Laser Micro/Nano-Fabrication Laboratory, School of Mechanical Engineering, Beijing Institute of Technology, Beijing, 100081, China

**Keywords:** depth measurement, double-helix beam, edge detection, metasurface

## Abstract

How to simultaneously obtain the depth, edge, and other light information of the scene to accurately perceive the physical world is an important issue for imaging systems. However, such tasks usually require bulky optical components and active illumination methods. Here, we design and experimentally validate a single geometric metasurface that can achieve depth measurement or edge detection under incoherent or coherent light respectively. Double helix point source function is utilized, and three verification experiments are carried out, including double-helix beam calibration, 2D object and 3D object detection, respectively. Additionally, two-dimensional edge detection can also be achieved. This compact imaging system can enable new applications in various fields, from machine vision to microscopy.

## 1 Introduction

Depth measurement and edge detection are essential tasks in many applications. Because edge information is a fundamental feature of an object, containing important geometric characteristics for various image processing tasks, which can be realized by filtering the lower spatial frequencies [1]. While for depth measurement, conventional cameras can only capture two-dimensional projections of intensity information from three-dimensional scenes without any knowledge of depth, but the rapidly-developing 3D imaging technology allows depth feature to be revealed by capturing extra light information [2, 3]. Both techniques are crucial to the operation of numerous applications, such as automatic driving [4, 5], artificial intelligence recognition [6], machine vision [7], and medical imaging [8, 9].

Recently, various approaches for depth measurement have been proposed, including stereo vision, time-of-flight, and point sources/fringe projection, but these often require active illumination or multiple viewpoints that increase the complexity of the system [10]. Alternatively, there are several methods that obtain depth information from a sequence of images under different defocus settings [11], but the precision is fundamentally limited because of the slowly changes of point spread function (PSF) with depth as well as ambiguities of direction of defocused distances. Hence, setting PSFs [12] which can achieve more precisely depth characterization is very important. Among them, the double-helix point spread function (DH-PSF) is a prominent example [11, 13], [14], [15], which produces a beam with two foci that rotate continuously in plane in response to the shifting distance of a point source.

Meanwhile, traditional edge detection methods usually utilize structured light to perform spatial differentiation for optical analog computing. However, some of the demonstrations on spatial differentiation are one-dimensional, which typically leads to anisotropic edge detection of the object and thus is not perfectly suited to imaging applications. Recently, the spiral phase provides a new way for edge detection [16]. Because the opposite halves of any radial line of the spiral phase can introduce a phase difference of *π* between the positive and negative spatial frequencies of incident light, which leads to a strong isotropic edge contrast enhancement of observed objects. However, the spatial light modulator (SLM) or the spiral phase plate [17, 18], which generates a spiral phase, makes the overall system too bulky and limits the resolution.

Optical metasurfaces provide promising platforms to develop unconventional ultrathin devices with multiple functionalities, which can meet the demand for system minimization and integration [19–23]. Metasurfaces have demonstrated unprecedented capabilities in manipulating light at the subwavelength scale, such as wavefront shaping [24–26], holography [27–29], secret sharing [30, 31], polarization control [32], and all-optical calculations and neural networks [33]. With regard to depth measurement, metalens array [34] forming light field camera have been used to demonstrate depth imaging. Meanwhile, three-dimensional imaging has also been achieved with two interleaved off-axis focusing metalenses utilizing spatial multiplexing [35]. While utilizing spatial differentiation approaches [8], optical edge detection can be achieved. An optical spatial differentiator based on the interference effects associated with surface plasmon excitations has been demonstrated. Besides, based on photonic crystal slab acting as a Laplacian operator that transforms an image into its second-order derivative, direct discrimination of the edges in the image can be achieved. However, previous reports are usually limited to performing only a single functionality in depth measurement or edge detection. And the detection systems that combine multiple functionalities tend to be bulky.

**Figure 1:**
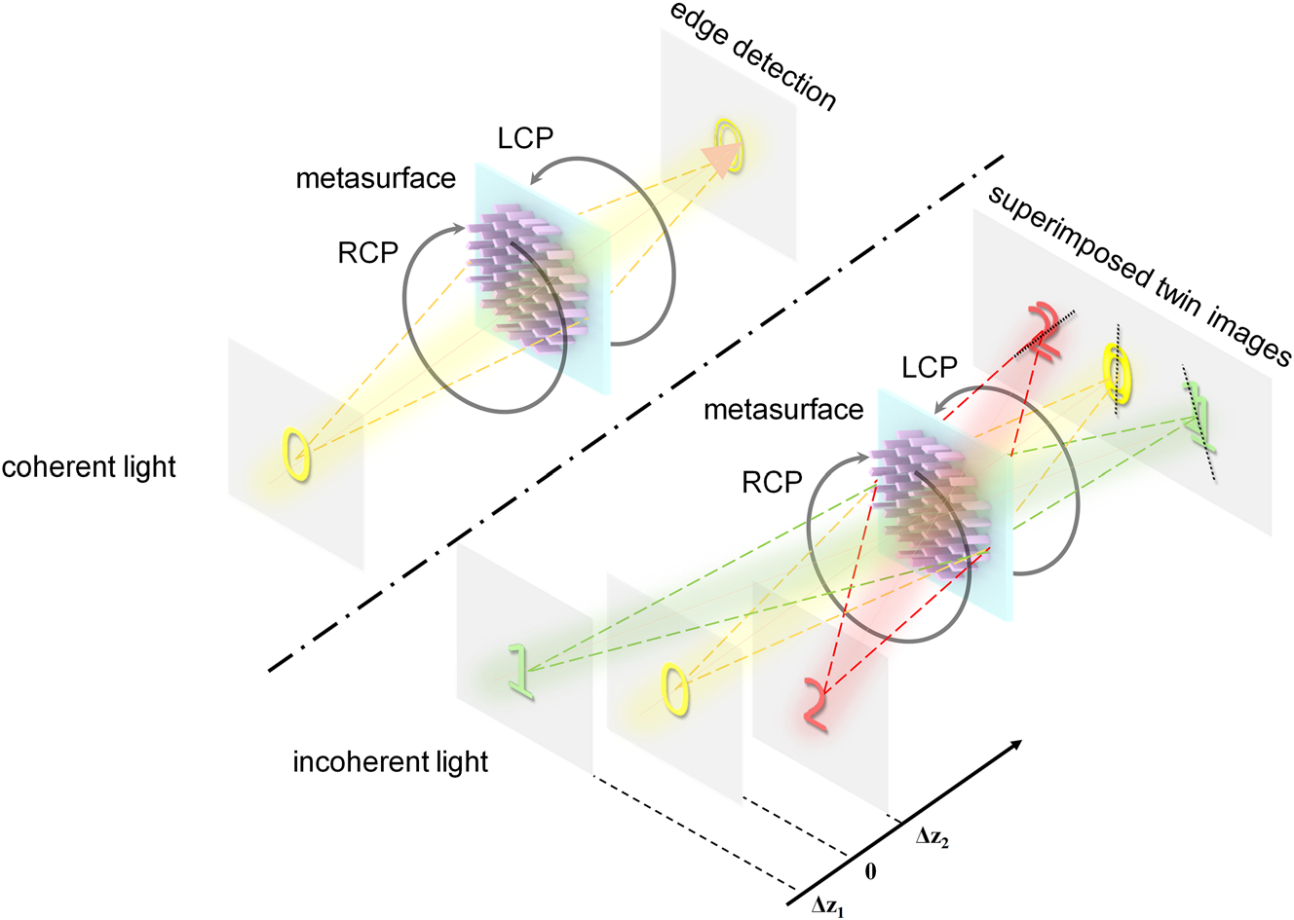
Schematic diagram of multifunctional metasurface for achieving edge detection and depth measurement. The system can extract depth information via the superimposed twin images with different rotation angles under incoherent light, and perform edge detection of objects under coherent light.

In this paper, we propose and develop a multifunctional metasurface by combining depth measurement and edge detection based on the double-helix point spread function method. As shown in Figure 1, an all-dielectric geometric metasurface encoded with DH-PSF phase is implemented in frequency domain. Such metasurface can generate DH-PSF with linear spiral rates at all spatial positions, while keeping the inter-lobe distance for these PSFs unaffected by defocusing. Under the illumination of incoherent light, the depth information of objects can be extracted by algorithmically analyzing the detected superimposed twin images with different rotations due to the convolution operation of object light and the DH-PSF. While under coherent light illumination, the central zone of the phase profile has the characteristic of spiral phase distribution, which can be utilized to achieve two-dimensional edge detection based on destructive interference. We experimentally characterize the optical response of such metasurface, as well as achieve depth and edge detection of both 2D object and 3D object at different locations under different illumination conditions. With its promising capabilities, such method may open new doors for machine vision, autonomous vehicles, and many other optical detection applications.

## 2 Principle and design

**Figure 2:**
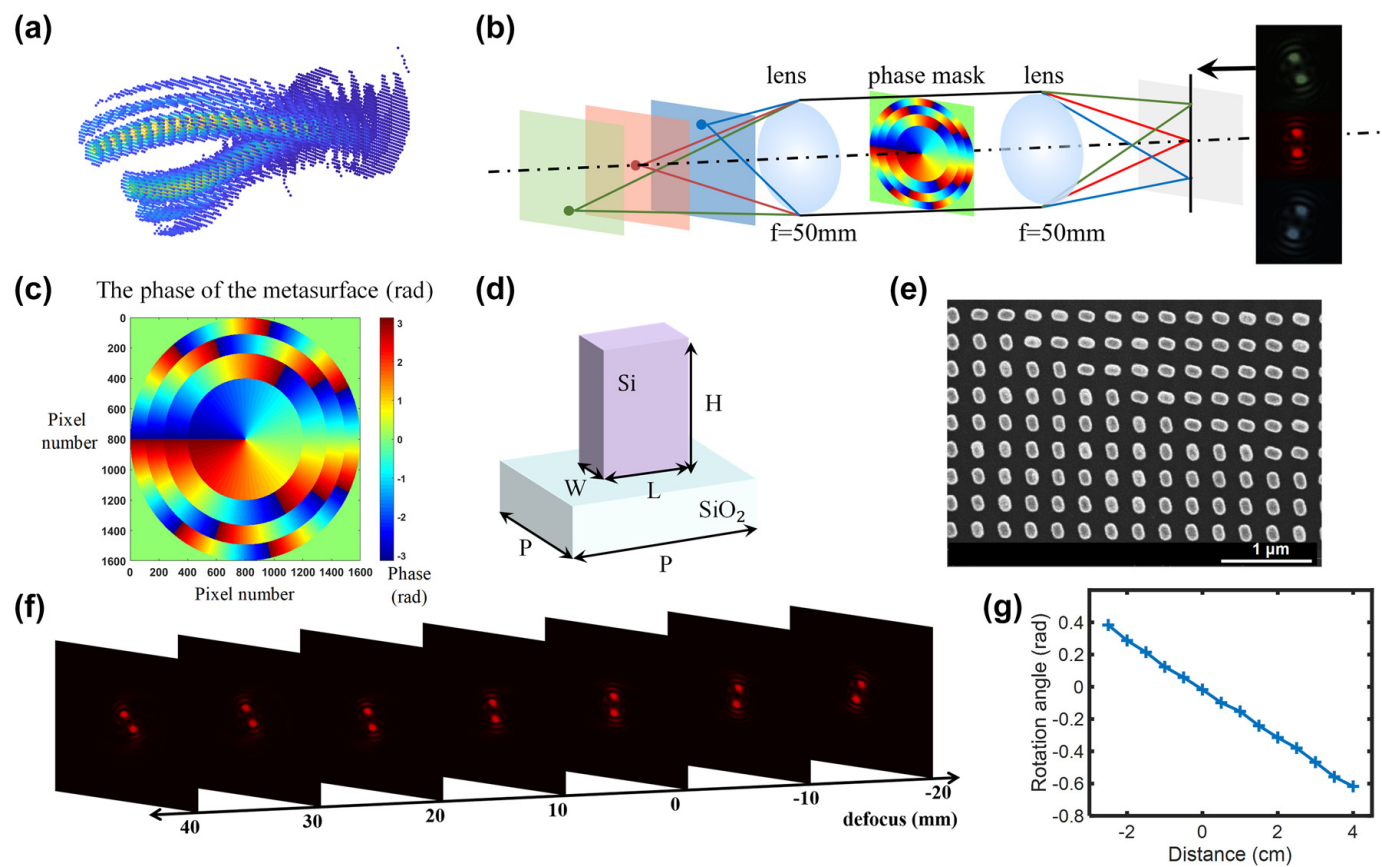
Double helix phase characteristics and the design and experimental calibration of the metasurface design. (a) DH-PSF has two lobes spinning around the optical axis along the direction of propagation. (b) Double helix point spread functions generated by a point light source at different depths through the phase modulation by the metasurface. (c) The phase distribution of the metasurface. (d) A schematic diagram of a single structure of the metasurface sample, its height *H* = 600 nm, period *P* = 300 nm, length *L* = 134 nm, and width *W* = 70 nm. (e) Scanning electron microscope (SEM) pattern of the metasurface sample. (f) Experimental diagram of double helix point spread function generated by point light sources at different positions passing through the metasurface. (g) The relation between the rotation angle of the DH-PSF and the positions of the point light sources.

The depth estimation solution proposed is based on the evolution of the double-helix PSF(DH-PSF) within three-dimensional space. As shown in Figure 2(a), the DH-PSF exhibits two lobes that spin around the optical axis along the direction of propagation. By convolution operation of such DH-PSF with object information, one can obtain the depth information accordingly. The whole optical system consists of a 4-f system with an additional metasurface phase mask placed on the frequency domain of it. As shown in Figure 2(b), when the light of a point source passes through such optical setup, the DH-PSF with distinct spatial rotation within three-dimensional space can be generated and distinct focal spots (main lobes) can be created on selected planes. As the point source moves back and forth near the focus, these two axisymmetric main lobes rotate around the center along the axis, and their rotation angle has an approximate linear relationship with the change of the axial position of the point source, which unambiguously encodes the depth information.

We have adopted the approach based on spiral phase (SP) for generating the phase profile which exhibits the DH-PSF. The DH-PSF generated has compact main lobes, and the rotation rate can be easily controlled by changing the number of the Fresnel zones. These excellent characteristics allow one to obtain an appropriate extended range and rotation rate of the DH-PSF by adjusting corresponding parameters. The phase profile we used to generate the DH-PSF can be directly expressed as:

(1) $$\begin{array}{ll} & P_{N}\left(\rho ,\varphi \right)\\ & \;=\left\{\begin{array}{c} \left(2n-1\right)\varphi ,R\sqrt{\frac{n-1}{N}}<\rho \leq R\sqrt{\frac{n}{N}},\;\;\;n=1,2,\ldots ,N\;\\ 0,\rho >R\; \end{array}\right. \end{array}$$

In this formula, *P*_*N*_(*ρ*, *φ*) is the phase at position (*ρ*, *φ*) under polar coordinate, and *R* represents the maximum radius. It can be observed that this particular phase is consist of a sequence of Fresnel zones, and *N* denotes the number of Fresnel zones. Each Fresnel zone corresponding to a spiral phase with a distinct topological charge, which increases in an arithmetic progression from the inside out with an addition of 2 for each layer. Here, considering the distinguishability of the DH-PSF and the depth measurement accuracy of the metasurface optical system, we choose *N* = 4 for phase generation, and the resultant phase distribution is demonstrated in Figure 2(c).

The complex amplitude *U* of a point source through interaction with such 4-f system integrated with metasurface can be expressed [36]:

(2) $$\begin{array}{c} U\propto 2\pi \overset{N}{\underset{n=1}{\sum }}i^{\left(2n-1\right)}\exp\left[\text{i}\left(2n-1\right)\varphi \right]*\\ \overset{R\sqrt{\frac{n}{N}}}{\underset{R\sqrt{\frac{n-1}{N}}}{\int }}\exp\left(-\text{i}\frac{k\Delta \phi }{2}\rho^{2}\right)J_{\left(2n-1\right)}\left(2\pi \rho F^{\prime }\right)\rho d\rho \end{array}$$

The parameters ΔΦ and *F*′ represent the defocusing and the vector of spatial frequencies, respectively, where

(3) $$\Delta \phi ={\left(\Delta z+\Delta z^{\prime }\right)/f}^{2}$$

(4) $$F^{\prime }=\rho^{\prime }/\left(\lambda f\right)$$

where Δ*z* and Δ*z*′ are the defocus distances of object and image, *f* is the focal length of the lenses in the 4-f system, *ρ′* represents the polar radius of each point at image plane, and *λ* represents the wavelength of the incident light. Jn represents Bessel function. By adjusting the number of the Fresnel zones, the rotation rate of the DH-PSF can be controlled simply and effectively. The specific expression of the DH-PSF defocus rotation rate can be expressed as follows:

(5) $$\frac{d\varphi }{d\Delta z}=\frac{\pi NA^{2}}{\lambda N\Delta l}$$

while NA denotes the numerical aperture of the experimental optical path. For a 4-f system which integrate such metasurface in the frequency domain, within a certain range, NA ≈ *R*/*f* is met. Additionally, Δ*l* refers to the difference in the topological charge for each layer, as mentioned above, Δ*l* = 2. As per the aforementioned equation, it is evident that the rotation rate of the DH-PSF changes with the number of Fresnel zones, which leads to an opposite change depth measurement extended range. Theoretically, we can observe that as the two main lobes of the DH-PSF rotate for 180°, the corresponding depth detection range can be calculated. In addition, due to the linear rotation rate of the spiral-phase-based PSFs at all axial positions, a more convenient and accurate conversion between the rotation angle and the depth can be achieved.

Based on the aforementioned principle, we took into account both efficiency and phase modulation and employed the geometric phase principle to design the metasurface. A schematic illustration of a singular structure from the metasurface sample is displayed in Figure 2(d). To optimize the meta-atoms, we swept the length, width, height and period of unit cell by using a rigorous coupled-wave analysis method. We chose amorphous silicon (α-Si) nanofins as building blocks, which behave as local half-wave plates and maximize circular cross-polarization conversion transmittance at the wavelength of 633 nm. Considering the balance of fabrication accuracy and broadband property, we ultimately opted for a 600 nm height nanorod with a length of 134 nm, a width of 70 nm, with a period of 300 nm in both the *x*- and *y*-directions. According to the geometric phase modulation mechanism, each nanofin induces an azimuthal-angle-dependent local abrupt phase change, which occurs for circularly polarized light when converted to its opposite helicity. We fabricated our sample on a glass substrate following silicon deposition, patterning, lift-off, and etching. The diagram of the sample as well as its SEM diagram are showcased in Figure 2(e).

In order to achieve depth measurement, we conducted experiments with DH-PSF engineered metasurface to obtain the specific changes of the rotation angle of the main lobes. As shown in Figure 2(f), during rotation, the size of the main lobes, the distance between the main lobes, and the sharpness of the main lobes are basically unchanged. In order to avoid additional restrictions brought by lens NA on the optical path system, a large-diameter lens with *f* = 50 mm was selected for our 4-f system. Furthermore, we experimentally calibrated the rotation angle of DH-PSFs and the corresponding distances from the point sources to the object focus, as shown in Figure 2(g). Through the check, when the distance between the point source and the object focus was within the range of the −25 mm–40 mm, the rotation angle has a range of 1 rad, the defocus rotation rate of the DH-PSF complies with the above Equation (3).

Meanwhile, the central phase profile of metasurface has the characteristic of spiral phase distribution, which can be utilized to achieve edge detection upon focused coherent light illumination. In this case, arbitrary two points located in symmetric positions to the origin have the same amplitude but out of phase *π*. When the light passing through, the target object is convolved with central part of DH-PSF, the monotonic phase/amplitude region is canceled by destructive interference, leading to a strong isotropic edge contrast enhancement of observed objects. The image quality of the edge detection results with higher topological charges becomes worse in comparison with *l* = 1 [37, 38], that is because the phase difference of two central asymmetric points is no longer *π*, which deteriorates the destructive interference. However, we actually did not block the Fresnel zones with higher topological charges in experiments, because the image quality degrading induced by them is tolerable.

## 3 Experimental and results

When a double-helix point spread function encoded metasurface is used to detect the depth information of object, the resulting superimposed images with rotation angles due to the convolution of object and DH-PSFs can incorporate axial position information. By analyzing the cepstrum of an image, one can extracting the rotation angle of the main lobe present in the detected images, and then ascertain the corresponding axial position details of the object by referring to the look-up table as shown in Figure 2(g).

**Figure 3:**
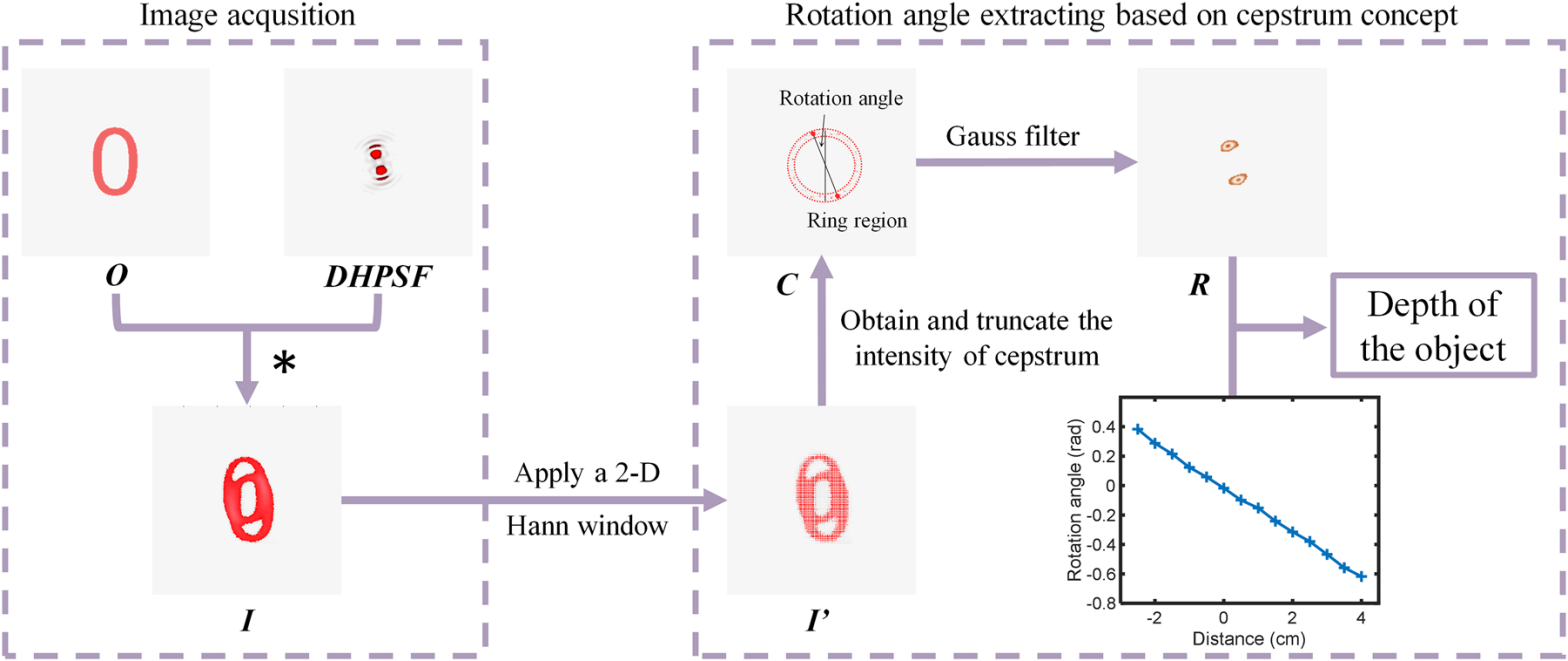
Depth extraction algorithm. The double helix point spread function is obtained through digital Fourier transformation of the acquired image. The rotation angle of the main lobe in the Fourier domain is calculated by using the two-dimensional fast Fourier transform and phase retrieval algorithm. The distance from the corresponding point light source to the focal plane in the object space can be calculated from the obtained rotation angle, which indicates the depth information of the object.

As shown in Figure 3, the object passing through the metasurface will be convolved with the DH-PSF, which resulting in superimposed twin images. The distance of different object points can be retrieved by numerically analyzing the image cepstrum and retrieving the local orientation of the DH-PSF [39]. In accordance with this method, before the cepstrum calculation, a two-dimensional Hann window is initially applied to the image in order to increase the reliability of the peak identification. The Hann window we used can be written as:

(6) $$I^{\prime }=W\left\{I\right\}=I\cdot \left\{\frac{1}{4}\left[1-\text{cos}\left(\frac{2\pi m}{S_{\text{w}}}\right)\right]\left[1-\cos\left(\frac{2\pi n}{S_{\text{w}}}\right)\right]\right\}$$

Where *m* and *n* are the spatial coordinates of the whole picture in both axes, and *S*_w_ is the size of the window. We analyze the cepstrum of the example image, which can be expressed as:

(7) $$C=C\left\{I^{\prime }\right\}:=F^{-1}\left\{\log\left({\left|F\left\{I^{\prime }\right\}\right|}^{2}\right)\right\}$$

where F represents Fourier transform. Then we extract the peak position present in the ring region which marked out by the dotted lines in ***C***. Based on the experimental outcomes presented in Figure 2(f), we extracted the distance and width of the double helix peak produced by the sample, and utilized them to truncate the cepstrum results. Afterwards, we perform Gaussian filtering on the truncated cepstrum ***C*** to mitigate the impact of noise on peak detection. Finally, by computing the peak rotation angle according to the established look-up table, we can determine the axial position information of the object.

**Figure 4:**
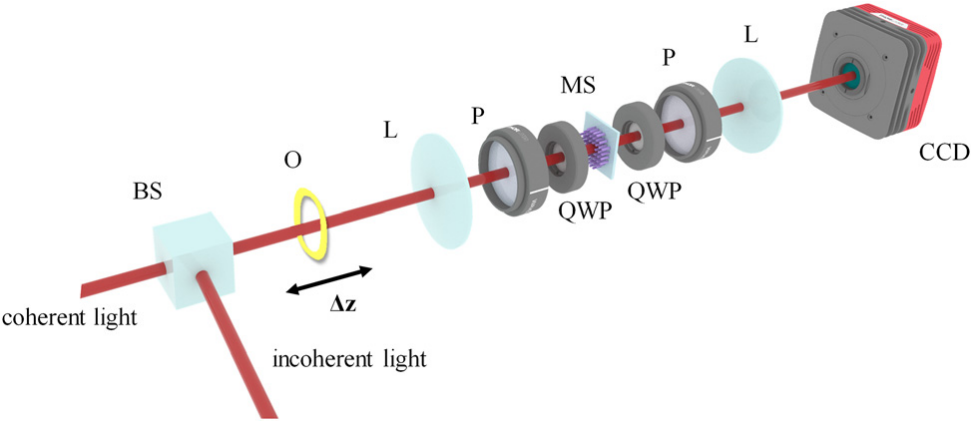
Optical setup of the multifunctional metasurface system realizing edge detection and depth measurement. BS: beam splitter, O: object, L: lens, P: linear polarizer, QWP: quarter waveplate, MS: metasurface.

To validate the performance of the sample, we employed the experimental setup illustrated in Figure 4. The metasurface is located in the frequency domain of a 4f system, by using two lenses with the focal length of 50 mm, the diameter of 50.8 mm, and their numerical apertures are 0.508. During the experiment, we set double light paths by using an incoherent light source of halogen lamp and coherent laser source, respectively. While two pairs of linear polarizer and quarter-wave plate are placed both before and after the metasurface, to select the corresponding circular polarization for ensuring the phase modulation. A narrow bandpass filter in front of the CCD, hence only the light with 633 nm wavelength can be collected. And the light passing through the object can hit the detection system accordingly.

**Figure 5:**
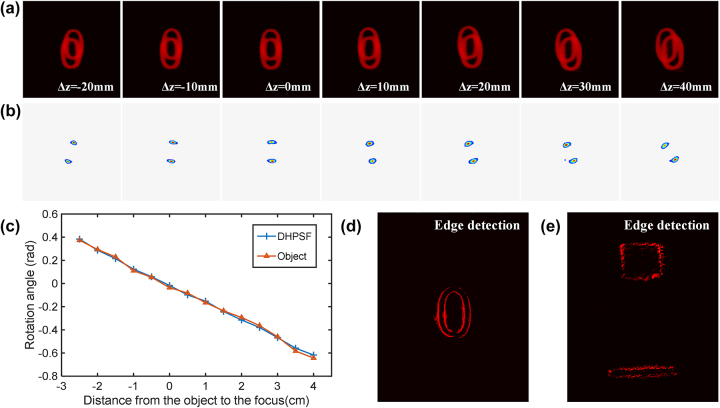
Results of edge detection and depth extraction of 2D object in different positions. (a) and (b) Imaging results of the 2D objects at different distances Δ*z* from the focal plane in the object space. (c) The rotation angles of the main lobe of the double helix function extracted from images with different depths are in good agreement with established look-up table. (d) and (e) Results of edge information of 2D and 3D objects, respectively.

First, we validate the capability of the metasurface to perform depth extraction by using a 2D plane image at different axial locations. During the experiment, we used the “0” on the resolution plate (group number “0” on GCG-020602 USAF1951 calibration board) as the object and adjusted its position before and after the focal plane in the object space. We then recorded the corresponding superimposed twin images and the distance Δ*z* from the object focus, as shown in Figure 5(a). By utilizing the depth extraction algorithm, we were able to extract the cepstrum information from the image and acquire the resulting outcome, as demonstrated in Figure 5(b). Using the above algorithm, through the calculation of the peak rotation angle of the captured images, we obtained the depth information of the moving object “0”, as shown in Figure 5(c). It is apparent that the extracted rotation angle of the main lobe present in the double helix function from captured images at varying depths exhibit robust agreement with the previously calibrations. The absolute value difference between the retrieved rotation angles and experimentally recorded ones is about 0.012 radians, which translates to a depth measurement that deviates from the actual value by ±2.6 mm, corresponding to a relative error of approximately 5 %. Hence, by leveraging image processing and the calibrated distance-angle relationship, we can attain the axial depth distances of objects situated at diverse locations.

Additionally, the metasurface is capable of performing edge detection under coherent laser source. At this time, the central part of the metasurface with the characteristic of spiral phase distribution plays a major role. When the incident coherent light interacts with the central part of the metasurface, the target object gets convolved with spiral phase gradient. The monotonous phase/amplitude regions are canceled by destructive interference, leaving only the highly-contrasted regions (edge of the object). Consequently, the edge information of object “0” can be acquired, as shown in Figure 5(d). Furthermore, the system is capable of detecting the edges of two objects located at different depths. We employed two distinct depths for imaging, where both objects (square and rectangular) were produced through 3D printing on a “Z-shaped” device with fixed depth of 10 mm, and the acquired edge detection results are presented in Figure 5(e).

**Figure 6:**
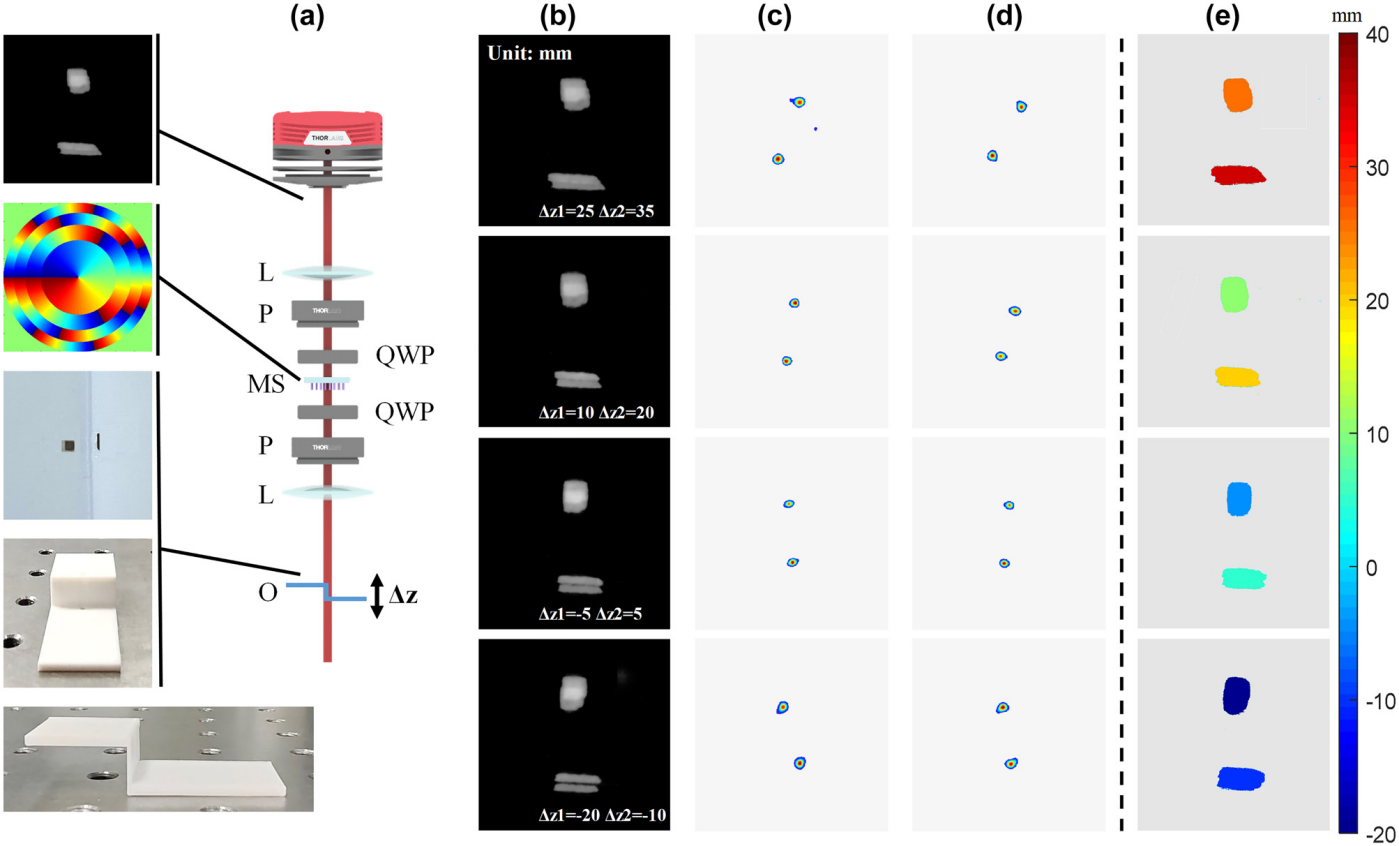
Validation of the depth detection of the metasurface. (a) Experimental optical setup for two objects with different axial locations. BS: beam splitter, O: object, L: lens, P: linear polarizer, QWP: quarter waveplate, MS: metasurface. (b) Imaging results of the objects at different distances Δ*z* from the focal plane in the object space. (c) and (d) The depth information of the upper square and lower rectangular bar. (e) Obtained depth map for objects in the field of view.

To further validate the depth detection capabilities of the metasurface, we performed another experiments utilizing two objects situated at distinct depths. Figure 6(a) presents the experimental optical path which resembled the incoherent light path demonstrated in Figure 4, with the two objects are substituted with a “Z-shaped” device located at different depths. During the experiment, we altered the front and rear positions of the “Z-shaped” device within the detection range and recorded the imaging results displayed in Figure 6(b). In this case, both objects were convolved with the DH-PSF, forming two pairs of superimposed images. By extracting the cepstrum information from the twin images obtained from each objects situated at varying depths, we were able to obtain the depth information of the upper square and lower rectangular bar, as represented in Figure 6(c) and (d), respectively. Note the relative separation of the two objects is fixed as 10 mm due to the location on “Z-shaped” setting. By computing the rotation angle of the convolved images, we derived the depth information illustrated in Figure 6(e) based on the established distance-angle correlation. We can conclude that for a real 3D object with multiple depths, we can segment the image and apply the same method for depth detection, to obtain the edge detection results of the 3D images.

## 4 Discussion and conclusion

To summarize, we propose and demonstrate a multifunctional integrated metasurface system for depth measurement and edge detection based on DH-PSF. In the case of incoherent light irradiation, we verified the orientations of the main lobes for the extracted DH-PSFs, which is in good agreement with them of the DH-PSFs generated by point light sources at different positions. Then, when objects of different depths are imaged through the metasurface in the 4f system, their depth information can be deduced by the rotation angles of the DH-PSFs obtained through the depth extraction algorithm. Furthermore, the system is capable of detecting the edges of objects under coherent light illumination, due to the central phase profile of metasurface has the characteristic of spiral phase distribution, which can induce destructive interference and then lead to a strong isotropic edge contrast enhancement of observed objects.

By exploiting the advantages of metasurface integration and miniaturization, such metasurface can be employed in a wider range of scenarios. For instance, with the use of multi-dimensional information acquisition techniques, we can obtain extra information including polarization, spectrum, depth or edge information to improve measurement and detection accuracy and efficiency. Simultaneously, efficient extraction algorithms are also key factors to better understand and process data, enabling us to effectively extract useful information in the practical applications. Furthermore, a multi-channel design could be implemented on the metasurface to endow the system with additional functionalities. In summary, such metasurface systems have broad application prospects and will play important roles in machine vision, microscopy, automatic driving, virtual and augmented reality, and so on.
